# Anilofos Tolerance and Its Mineralization by the Cyanobacterium *Synechocystis* sp. Strain PUPCCC 64

**DOI:** 10.1371/journal.pone.0053445

**Published:** 2013-01-31

**Authors:** D. P. Singh, J. I. S. Khattar, Mandeep Kaur, Gurdeep Kaur, Meenu Gupta, Yadvinder Singh

**Affiliations:** Department of Botany, Punjabi University, Patiala, Punjab, India; Belgian Nuclear Research Centre SCK/CEN, Belgium

## Abstract

This study deals with anilofos tolerance and its mineralization by the common rice field cyanobacterium *Synechocystis* sp. strain PUPCCC 64. The organism tolerated anilofos up to 25 mg L^−1^. The herbicide caused inhibitory effects on photosynthetic pigments of the test organism in a dose-dependent manner. The organism exhibited 60, 89, 96, 85 and 79% decrease in chlorophyll a, carotenoids, phycocyanin, allophycocyanin and phycoerythrin, respectively, in 20 mg L^−1^ anilofos on day six. Activities of superoxide dismutase, catalase and peroxidase increased by 1.04 to 1.80 times over control cultures in presence of 20 mg L^−1^ anilofos. Glutathione content decreased by 26% while proline content was unaffected by 20 mg L^−1^ anilofos. The test organism showed intracellular uptake and metabolized the herbicide. Uptake of herbicide by test organism was fast during initial six hours followed by slow uptake until 120 hours. The organism exhibited maximum anilofos removal at 100 mg protein L^−1^, pH 8.0 and 30°C. Its growth in phosphate deficient basal medium in the presence of anilofos (2.5 mg L^−1^) indicated that herbicide was used by the strain PUPCCC 64 as a source of phosphate.

## Introduction

Weeds are a nuisance in field crops as they compete for soil and water resources, reduce crop quality, interfere with irrigation and harvesting operations, and reduce land value. The sole price of agricultural products gets lowered when weed seeds get mixed with harvested crops, which require extra cleaning operations. Besides this, weeds also serve as alternate host to many pathogens, insects and nematodes, resulting in increased costs of crop protection operations [1]. It is estimated that weeds account for 45% of the total annual loss of agricultural production from various pests in India [2].

Large scale use of herbicides that selectively kill weeds is an integral component of modern agriculture since these chemicals reduce labour and machine requirements. Discovery of 2,4-dichlorophenoxy acetic acid after second world war revolutionized agriculture in developed countries [1]. Since then, the use of herbicides has increased enormously across the world. In India, 240 pesticides are registered for use, out of which 51 are herbicides [3].

The herbicide use in India increased from 15 to 11000 tonnes during 1970 to 2002 [1]. The average consumption of herbicide in India is 60 g per hectare which accounts for 13% of the total global consumption (46% of pesticides). Punjab and Haryana are the leading states in India in terms of herbicide consumption [1]. Although herbicides helped the farmer community by increasing the crop yields, their use also resulted in a number of problems i.e. contamination of soil and water [4], [5], [6], [7], [8], negative effects on non-target organisms, such invertebrates [9], [10], [11], [12], fish [13], [14], amphibians [15] and microorganisms, including cyanobacteria [16], [17], [18], [19].

Cyanobacteria are an integral component of paddy field ecosystems where they play an important role in building of and maintaining soil fertility [20], [21], [22]. In the soil, they are reported to improve organic content, water holding capacity, nitrogen status, and release vitamins, plant stimulating hormones, extra cellular polysaccharides and also solubilize phosphates [23], [24]. More than half of the total nitrogen used in paddy crop derives from the native soil nitrogen pool which is maintained through biological nitrogen fixation by both hetero- and autotrophs in soil [25]. Thus, use of cyanobacterial biofertilizer is considered to be a good management of paddy fields since their use not only increases fertility of the soil but is also eco-friendly. The utilization of cyanobacterial biofertilizer in paddy fields requires that strains be tolerant or able to degrade a variety of routinely used agrochemicals, including herbicides.

The earlier work on the influence of herbicide on cyanobacteria has been extensively reviewed [26], [27]. Herbicides cause deleterious effects on cyanobacteria by influencing their growth, photosynthesis, nitrogen fixation, biochemical composition and metabolic activities [28]–[30]. Studies on the cyanobacterial tolerance to herbicides and the degradation of these herbicides are available in literature [31]–[33].

Anilofos (S-[2-[(4-chlorophenyl) (1-methylethyl) amino]-2-oxoethyl] O O-dimethyl phosphorodithioate) is the active chemical of the commercial grade herbicide ANILOGUARD (Gharda Chemical Limited, Ratangiri, Maharashtra, India). It belongs to the organophosphate group and is widely used as a pre-emergence and early post-emergence herbicide for the control of annual grasses, sedges and some broad-leaved weeds in transplanted and direct seeded rice crops. The herbicide is mixed with dry sand and is spread in the standing water after paddy plantation at the rate of 300 to 450 g active ingredient per hectare. Anilofos has 5 to 25 days (d) half-life in water flooded soil [34].

We have previously shown that *Synechocystis* sp. strain PUPCCC 64 is able to degrade chlorpyrifos [19]. In the present investigation we aim to understand the mechanism of anilofos tolerance and its degradation potential by this cyanobacterium strain so that it might be used in Indian biofertilizer technology programmes.

## Materials and Methods

### Microorganism and Culture Conditions

The cyanobacterium *Synechocystis* sp. strain PUPCCC 64, was isolated from rice fields of village Derabassi (30^o^ 58′ 72″ N; 76^o^ 8′ 28″ E) of district Mohali, Punjab [19]. It was grown in slightly modified Chu-10 medium [35] supplemented with micronutrients [36]. One liter nutrient medium contained 0.232 g CaCl_2_.2H_2_O, 0.025 g MgSO_4_.7H_2_O, 0.02 g Na_2_CO_3_, 0.044 g Na_2_SiO_3_.5H_2_O, 0.01 g K_2_HPO_4_, 0.0035 g of ferric citrate and citric acid each and 1 g, KNO_3_. Cultures were maintained in a room at 28±2°C and illuminated 14 h daily with a light intensity of 44.5 µE (µmoles of photons m^−2^ s^−1^). Exponentially growing cultures (8 d old) were used throughout our study and each experiment was repeated three times.

### Growth of *Synechocystis* sp. Strain PUPCCC 64 in Anilofos

The tolerance limit of *Synechocystis* sp. against herbicide was determined by growing it in graded concentrations of anilofos (5–30 mg L^−1^). These concentrations were prepared in 250 mL Erlenmeyer flasks containing 100 mL Chu-10 medium from the stock solution of commercial grade ANILOGUARD (30% Emulsifiable Concentrate). After two washings with sterilized double distilled water, exponentially growing stock cultures were inoculated in anilofos containing media to get the initial absorbance of 0.1 at 680 nm. At regular intervals of 2 d, extending up to 12 d, 10 mL samples were withdrawn and growth was measured spectrophotometrically as the increase in absorbance of the cultures with a Thermo Spectronic model 20D+ (Thermo Spectronic, Rochester, NY).

### Biochemical Analysis

#### Photosynthetic pigments

Cyanobacterial suspensions (10 mL) were centrifuged at 5000 rpm, washed thrice with double distilled water and a same volume of acetone (80%) was added to the cell pellet. The mixture was shaken vigorously and kept at room temperature for 12 h to extract acetone soluble photosynthetic pigments. The extracts were centrifuged at 5000 rpm, attained the same volume of supernatant by adding sufficient amount of acetone and absorbance was checked at 660, 645 and 450 nm. The amount of chlorophyll a (Chl a) was calculated according to Holm [37] whereas total carotenoids (Car) were quantified as per Myers and Kratz [38].

Phycobiliproteins (PBS) were extracted in phosphate buffer (50 mM, pH 7.0) by freeze-thawing as described elsewhere [39]. Pellet of cyanobacterial cells obtained after centrifugation at 5000 rpm were washed with double distilled water and suspended in the same buffer containing lysozyme (1 g L^−1^). The mixture was incubated at 37°C in a water bath for 1 h with continuous stirring. Obtained spheroplasts were gently washed by centrifugation at 3000 rpm and resuspended in phosphate buffer. The resultant solution was then subjected to 10–12 freeze-thawing cycles until all the pigments were released from the cells. After centrifugation at 5000 rpm, the absorbance of the supernatant was recorded at 565, 615 and 652 nm.

#### Antioxidant enzymes

Superoxide dismutase (SOD) activity was measured as inhibition of reduction of nitroblue tetrazolium chloride (NBT) photochemically [40]. Thick cell suspension, obtained by centrifugation at 5000 rpm, was washed twice with double distilled water, resuspended in a small volume of 100 mM phosphate buffer (pH 7.8) and disintegrated with a sonicator (Soniprep 150, Sanyo, USA), by giving 5 pulses (5 micron amplitude) each of 1 min, with 30 s intervals. Supernatant obtained after centrifugation (15,000 rpm for 20 min, at 4°C) was used as an enzyme extract. The assay mixture for the estimation of SOD was prepared by mixing together 27 mL of 100 mM sodium phosphate buffer (pH 7.8), 1.5 mL of methionine (3%), 1 mL of NBT (0.014%), 1 mL of Na_2_CO_3_ (23.4%) and 1.5 mL of EDTA (2 mM). To 2.7 mL of reaction mixture, 0.1 mL enzyme extract and 0.2 mL of riboflavin (0.015%) were added. After mixing, test tubes were illuminated for 5 min under fluorescent lights (44.5 µE). The reaction was terminated by transferring the reaction mixture to the dark. Tubes containing reaction mixture with enzyme extract served as blank and were kept in the dark. Control tubes without enzyme extract served as controls and were kept in the light. The absorbance of solution was measured at 560 nm. One unit of SOD activity is the amount of enzyme required to inhibit the reduction of NBT in light by 50%.

Peroxidase (POD) activity was measured according to the method of Gahagen et al. [41]. The reaction mixture contained 2.0 mL double distilled water, 0.35 mL phosphate buffer (100 mM, pH 6.8), 0.2 mL of 0.5% H_2_O_2_, 0.3 mL of 5% pyrogallol and 0.15 mL of enzyme extract. The increase in absorbance at 420 nm was measured up to 5 min after mixing the contents by inversion.

Catalase (CAT) activity was determined by measuring O_2_ release from the dissociation of H_2_O_2_ in darkness [42]. Three mL of 50 mM phosphate buffer (pH 7.0) containing 50 mM H_2_O_2_ were directly added to 1 mL cell suspension in the reaction vessel kept in dark. Oxygen released due to enzymatic dissociation of H_2_O_2_ was measured for 5 min using oxygen analyzer (YSI, USA). One unit of catalase is the amount of enzyme required to produce one nmol O_2_ min^−1^.

#### Glutathione and proline contents

Glutathione content (GSH) was determined according to the modified Ellman’s method [43]. Ten mL of cell suspension was centrifuged (5000 rpm for 10 min at 28°C), washed twice with double distilled water and sonicated in 5 mL of 0.2 M phosphate buffer (pH 8.0) for 30 min. The supernatant obtained after centrifugation at 20,000 rpm for 20 min and 4°C was used for estimation of GSH. The reaction mixture was prepared by adding 0.2 mL of enzyme extract, 3 mL of phosphate buffer (pH 8.0) and 0.5 mL of 5–5′-dithiobis-2-nitrobenzoic acid. The development of colour intensity was measured at 412 nm. GSH was expressed in µg mg^−1^ protein. A standard curve was prepared by using GSH.

Proline content was measured according to the method of Bates et al. [44]. Twenty mL of culture was centrifuged at 5000 rpm, washed with double distilled water, resuspended in 10 mL of 3% aqueous sulphosalicylic acid, sonicated for 3–5 min and centrifuged at 20,000 rpm for 20 min at 4°C. Two mL of extract were taken in a test tube and 2 mL each of glacial acetic acid and acidic ninhydrin were added. The mixture was heated in a water bath at 100°C for 1 h and the reaction was stopped by shifting test tubes to an ice-bath. Then, 4 mL toluene was added to the reaction mixture. After stirring for 20–30 s, the toluene layer was separated and warmed to room temperature. Absorbance was measured at 520 nm. A standard curve was prepared using proline.

### Anilofos Removal and Mineralization by *Synechocystis* sp. PUPCCC 64

Uptake experiments were conducted in 250 mL Erlenmeyer flasks containing 100 mL medium supplemented with 10 mg L^−1^ anilofos (concentration inhibiting 20% growth). Exponential growing cultures were harvested, washed twice with double distilled water and inoculated to obtain an initial absorbance of 0.5 at 680 nm. At regular intervals, 10 mL of cyanobacterial cells was withdrawn and centrifuged. For the estimation of intracellular herbicide and its degradation product(s) cells treated with anilofos for 4 days were harvested and sonicated in Chu-10 medium as described above in section antioxidant enzymes. Anilofos and its product(s) were extracted with hexane dichloromethane partitioned thrice [19]. Anilofos removal was optimized by studying its removal by varying cultural conditions i.e. pH (5.0–9.0) of medium, biomass load (25–200 mg L^−1^ protein), and temperature (20–40°C). At a time, one parameter was varied keeping others (optimized) constant.

#### Quantification of anilofos

Anilofos was quantified by HPLC (Waters, USA) injecting 20 µL of extracted sample of herbicide in acetonitrile. HPLC operating parameters were: column: C-18; detector: Ultra Violet-Visible; running phase: 70% acetonitrile; flow rate 0.5 mL min^−1^; running time 7 min.

### Anilofos as a Source of Phosphate

Three 250 ml Erlenmeyer flasks, each containing 100 mL Chu-10 medium with phosphate (0.01 g L^−1^), minus phosphate, and minus phosphate plus 2.5 mg L^−1^ anilofos were inoculated with washed cyanobacterial suspension to attain the initial absorbance of 0.5 at 680 nm. At regular interval of 2 d extending up to 12 d, 10 mL of cultures were withdrawn and absorbance was measured at 680 nm.

Protein content was determined according to Lowry et al. [45].

### Chemicals

All chemicals used in media preparation and enzymatic assays were obtained from Merck, India. Commercial grade anilofos (ANILOGUARD 30% w/v) was from Gharda Chemicals Limited, Ratangiri, Maharashtra, India.

### Statistical Analysis

All the data are the average of three independent experiments ± Standard Deviation (SD). Data were statistically analyzed by applying one way analysis of variance and Tukey’s honest significance difference test. All statistical analyses were tested against the probability value at 95% confidence level (p<0.05) using GraphPad Prism 6.0 version 6.0 (www.graphpad.com).

## Results and Discussion

### Growth of *Synechocystis* sp. Strain PUPCCC 64 in the Presence of Anilofos

**Figure 1:**
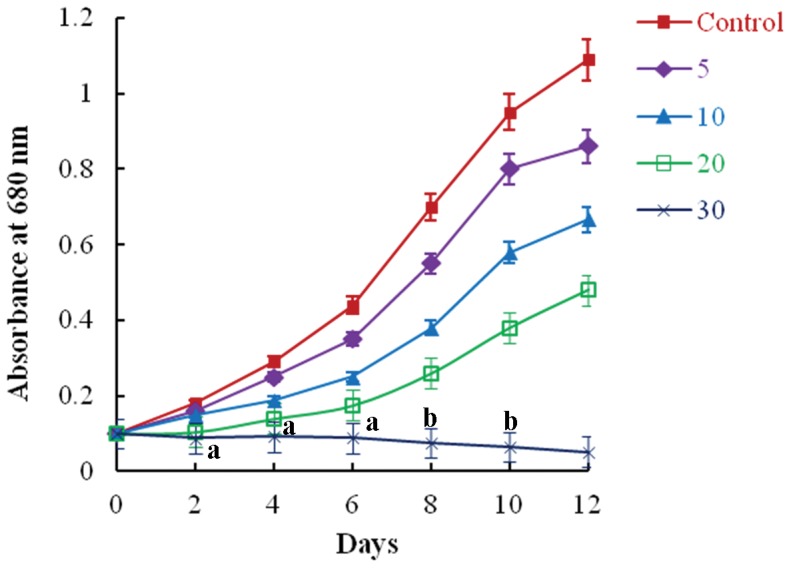
Growth of *Synechocystis* sp. strain PUPCCC 64 in basal medium supplemented with anilofos (mg L ^−**1**^ **).** All presented data are the mean values of three independent experiments ± SD Data at different time intervals with same lower case letters are not significantly different from each other at the 95% confidence level (p<0.05).

**Figure 2:**
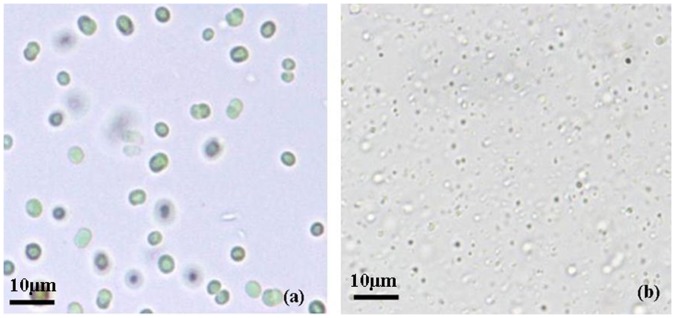
Photomicrograph of *Synechocystis* sp. strain PUPCCC 64 culture on day 4. (a): control; (b): anilofos (30 mg L^−1^).

The growth of cyanobacterial cells in graded concentrations (5–30 mg L^−1^) of anilofos is shown in Fig. 1. The growth of control cultures increased from 0.1 on zero day to 1.09 on 12 day. In the presence of anilofos, a concentration-dependent decreased growth was observed. The cyanobacterium survived in anilofos up to 20 mg L^−1^ and did not grow in 30 mg L^−1^ anilofos. In this concentration of anilofos, the cells lysed, pigments were released in the medium and cultures became colourless (Fig. 2). On day 12, the microorganism exhibited 61, 44 and 25% growth as compared to control cultures in 5, 10 and 20 mg L^−1^ of anilofos, respectively. Growth inhibition by anilofos in *Anabaena torulosa* [46], butachlor, bensulfuron-methyl in *Nostoc* sp. [47], [48], glyphosate in *Anabaena* sp., *Leptolyngbya boryana, Nostoc punctiforme,* and *Microcystis aeruginosa* [31], [49], and, molinate and bentazon in *Anabaena cylindrica* and *Nostoc muscorum* [29], [50], [51] has recently been reported. These herbicides affect photosynthesis (bentazon), cell division and inhibition of synthesis of long chain fatty acids (anilofos, butachlor and molinate), the shikimate pathway (glyphosate), and acetolactate synthase (bensulfuron-methyl) of target organisms [52].

### Photosynthetic Pigments

The presence of anilofos caused an inhibitory effect on the growth of *Synechocystis* sp. strain PUPCCC 64 in a concentration-dependent manner. On day six, the microorganism exhibited 21, 41 and 60% decrease in chlorophyll a (Chl a) content over control cultures with 5, 10 and 20 mg L^−1^ anilofos, respectively, whereas carotenoid (Car) content decreased by 32, 64 and 89%, respectively (Table 1). Phycocyanin (PC) content decreased by 55, 85 and 96% compared to control cultures with 5, 10 and 20 mg L^−1^ of anilofos, respectively, on 6 d. Allophycocyanin (APC) content decreased by 25, 75 and 85% while phycoerythrin (PE) contents were decreased by 47, 50 and 79% with 5, 10 and 20 mg L^−1^ of anilofos, respectively, on day six. Decreasing order of production of photosynthetic pigments by anilofos on day six was: PC>Car>APC>PE>Chl a. It is interesting to note that with 5 mg L^−1^ anilofos, which is 10 times the field concentration, levels of photosynthetic pigments decreased in the range of 21–55% (Table 1). Growth inhibition of PUPCCC 64 strain with increase of anilofos concentration can be explained on the basis of damaging effects of herbicide on photosynthetic pigments. Butachlor, bensulfuron-methyl and dimethoate inhibited photosynthetic pigments of *Nostoc* [48]. Treatment with bentazone (2 mM) for 72 h decreased total phycobiliproteins content of *Anabaena cylindrica* by 58% whereas molinate at highest tested concentration (2 mM) completely suppressed phycobiliproteins after 24 h treatment [29]. Anilofos inhibited all photosynthetic pigments of *Anabaena torulosa* [46] and *Oscillatoria simplicissima* [33]. Herbicides such as 3-(3,4-dichlorophenyl)-1,1-dimethyl urea (DCMU), 2-tert-butylamino-4-ethylamino-6-methylthio-1,3,5-triazine (terbutryn) and 4-hydroxy-3,5-diiodobenzonitrile (ioxynil) have been shown to inhibit PSII of photosynthetic organisms [53]–[56]. It has been shown that herbicides bind to the Q_B_ site of D1 subunit of PSII and block electron transfer from Q_A_ to Q_B_ [53], [55]–[56]. This has been evidenced by studying the crystal structure of PSII of *Thermosynechococcus elongatus* [56] and by causing mutations in the proteins binding to PSII subunits of cyanobacteria [54]–[55]. Mutational studies at the acceptor side of PSII have identified the role of D1-Ser264 in herbicide binding especially triazines [57]. Herbicides are mainly responsible for inhibiting PS II but Anilofos has also been reported to cause inhibition in cell division and synthesis of long chain fatty acids [52]. The present study suggests that anilofos caused inhibition of synthesis/degradation of photosynthetic pigments. To elucidate the exact mechanism of anilofos inhibition in *Synechocystis* sp. strain PUPCCC 64, additional studies are needed.

**Table 1:** Amount of photosynthetic pigments (mg L^−1^) of *Synechocystis* sp. strain PUPCCC 64 in anilofos-grown cultures at day 6.

Pigment	Anilofos (mg L^−1^)			
	Control	5.0	10	20
**Chl a**	1.2±0.05	0.94±0.01	0.71±0.01	0.48±0.01
**Car**	5.6±0.25	3.8±0.04	2.0±0.02	0.6±0.01
**PC**	20±0.2	9.0±0.15	3.0±0.01	0.8±0.01
**APC**	4.0±0.05	3.0±0.01	1.0±0.01	0.6±0.01
**PE**	16±0.1	8.5±0.05	8.0±0.02	3.3±0.02

Data in rows are significantly different from each other at the 95% confidence level (p<0.05).

Data are means of three observations ± SD.

Chl a: Chlorophyll a; Car: Carotenoid; PC: Phycocyanin; APC: Allophycocyanin; PE: Phycoerythrin.

### Antioxidant Enzymes

Toxicity of herbicides may lead to the generation of free radicals and cyanobacteria may respond to this stress by inducing antioxidant enzymatic defense mechanism. Superoxide dismutase (SOD) is involved in the neutralization of highly reactive oxygen species (ROS) such as superoxide radicals and singlet oxygen resulting in the generation of the lesser toxic hydrogen peroxide (H_2_O_2_) that is still harmful to cells [58] requiring removal by catalase (CAT) and/or peroxidase (POD) enzymes. Thus, the activity of these antioxidant enzymes in the PUPCCC 64 strain grown in anilofos for 24 h was studied.

**Figure 3:**
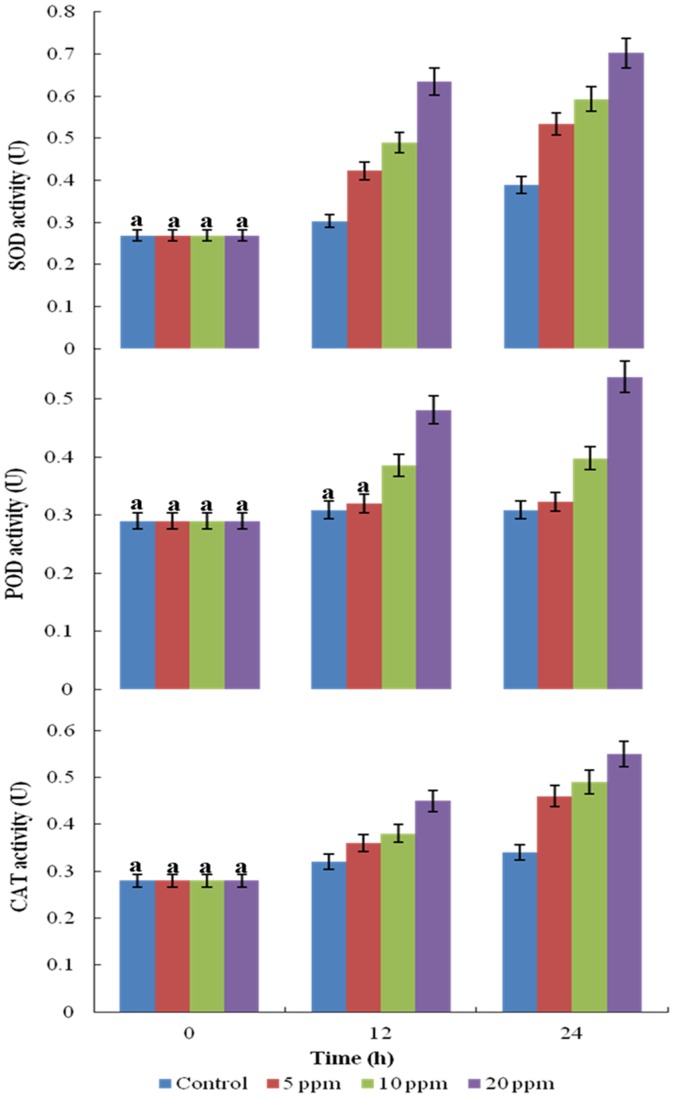
Activity of SOD, POD and CAT of *Synechocysti*s sp. strain PUPCCC 64 in presence of anilofos (mg L ^−**1**^ **).** All presented data are the mean values of three independent experiments ± SD Data at different time intervals with same lower case letters are not significantly different from each other at the 95% confidence level (p<0.05).

SOD, POD and CAT activities were stimulated significantly with higher anilofos concentration (Fig. 3). The cyanobacterium showed 137, 152 and 180% activities of SOD in 5, 10 and 20 mg L^−1^ of anilofos, respectively, at 24 h compared to control without herbicide (Fig 3a). POD activity was increased by 104, 128 and 174% (Fig. 3b) whereas activity of CAT increased by 109, 117 and 131% (Fig 3c) in 5, 10 and 20 mg L^−1^ anilofos compared to controls, respectively, at 24 h. The results showed that these enzymes played a key role in overcoming the oxidative stress caused by anilofos. The increase or decrease in level of antioxidant enzyme in cyanobacteria depends upon the type of organism or the nature of herbicides. The increase of antioxidant enzymes by anilofos (1.5–20 mg L^−1^) stress has been reported in cyanobacteria *A. torulosa* [46] and *O. simplicissima* [33]. Other herbicides such as glufosinate [59] and paraquat [60], at 0.5 and 0.75 µM, respectively, increased SOD and CAT activities by 3–4 times over control cultures in the unicellular green alga *Chlorella vulgaris*. Molinate decreased antioxidant enzymes (SOD, CAT and POD) and increased levels of lipid peroxidation leading to electrolyte leakage in the diazotrophic cyanobacterium *Nostoc muscorum* [51]. The increased activities of SOD, POD and CAT in the PUPCCC 64 strain, on the other hand, indicate that anilofos stress may have stimulated the generation of reactive oxygen species which were scavenged by the elevated levels of these enzymes and helped the organism to tolerate herbicide stress.

### Glutathione and Proline Contents

**Figure 4:**
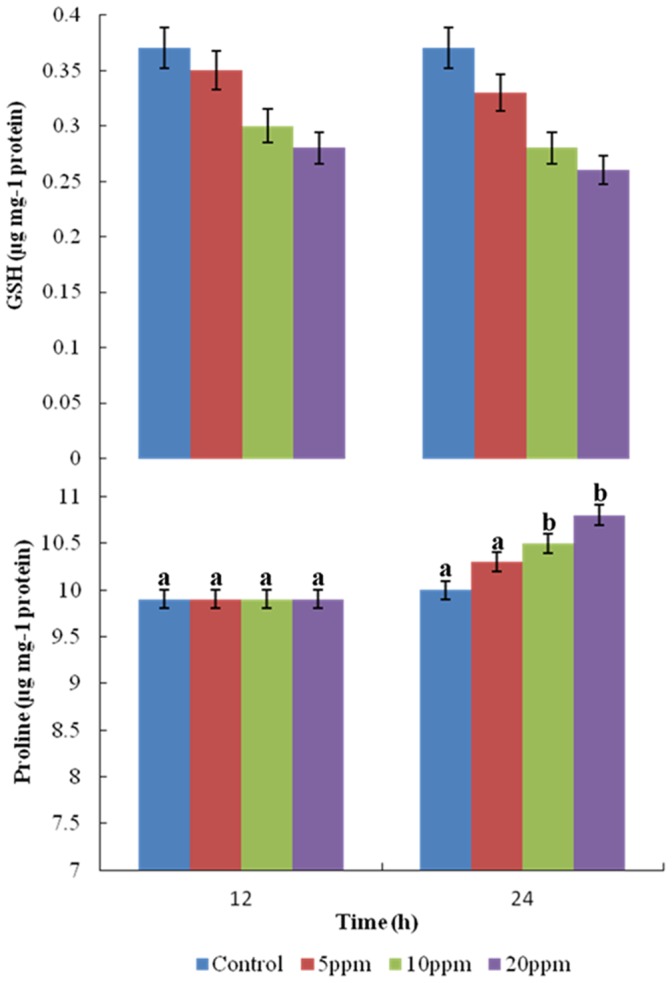
Effect of anilofos (mg L^−1^) on GSH and Proline content of *Synechocystis* sp. strain PUPCCC 64. All presented data are the mean values of three independent experiments ± SD Data at different time intervals with same lower case letters are not significantly different from each other at the 95% confidence level (p<0.05).

Low molecular weight compounds like GSH and proline also play a key role to counter abiotic stress in microorganisms and plants. Cellular GSH content of the PUPCCC 64 strain significantly decreased by 19 and 24% with 10 and 20 mg L^−1^ of anilofos, respectively. (Fig. 4a). A similar observation was reported in a rice field cyanobacterium *Nostoc muscorum* for which the GSH content decreased with an increase of 0.75 to 2.0 mM of molinate [51]. Glutathione is known to help in lowering the levels of H_2_O_2_ through the action of glutathione peroxidase [58]. Lowering of glutathione in strain PUPCCC 64 in presence of anilofos indicates that it may be helping in reducing the levels of H_2_O_2._

Proline accumulation under stress conditions is either due to its increased synthesis or decrease in degradation [61], [62]. Proline content did not significantly increase with 5 mg L^−1^ anilofos and only increased by 12% with 20 mg L^−1^ anilofos. (Fig. 4b). Thus, proline levels in strain PUPCCC 64 appeared to be unrelated to the organism’s herbicide tolerance. Similar observations were made for other cyanobacteria [63], [64] and it is unlikely that, of the two non-enzymatic defence systems, proline levels are related to herbicide-induced stress.

### Anilofos Removal

**Figure 5:**
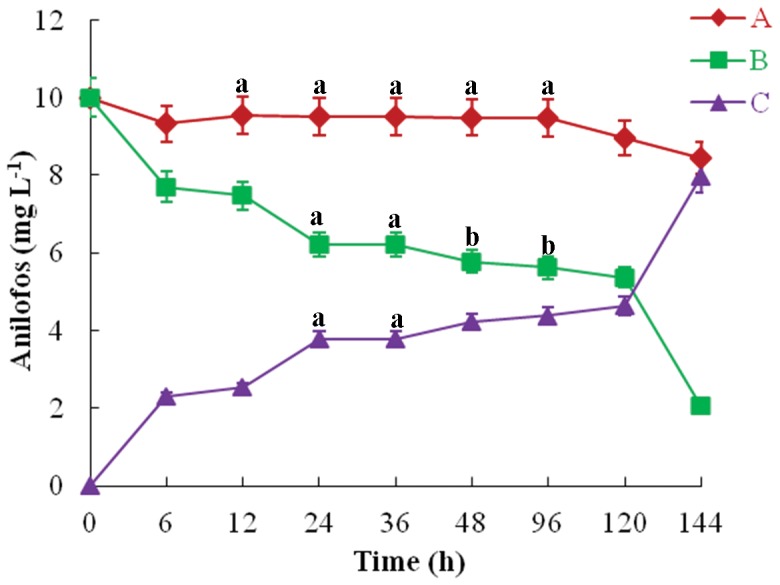
Uptake of anilofos by *Synechocystis* sp. strain PUPCCC 64. (A) control (without PUPCCC 64 cells); (B) anilofos in medium; (C) anilofos uptake All presented data are the mean values of three independent experiments ± SD Data at different time intervals with same lower case letters are not significantly different from each other at the 95% confidence level (p<0.05).

Anilofos removal by the PUPCCC 64 strain was studied to test whether the microorganism was able to take up and degrade the herbicide intracellularly. The organism showed continuous uptake of herbicide intracellularly up to six days (Fig. 5). The results revealed that the removal of anilofos was rapid during the initial hours (2.3 mg L^−1^ in 6 h) and afterwards removal slowed down up to 4 d. The cyanobacterium removed 4.2 and 7.9 mg L^−1^ anilofos in 4 and 6 d, respectively. It appears that anilofos is initially adsorbed on the surface of biomass followed by slow intracellular uptake. Orús and Marco [65] reported removal of trichlorfon from culture media supplemented with 300 mg L^−1^ of insecticide by *Gloeothece* PCC 6501, *Plectonema calothricoides*, *Anabaena* PCC 7119, *Nostoc* UAM 2005 and *Chlorogloeopsis* PCC 6912. Rapid uptake of the herbicides such as atrazine and terbutryn has been reported for the cyanobacterium *Synechococcus elongatus* and green alga *Chlorella vulgaris* [66]. Fast removal of chlorpyrifos in the initial 12 hours by *Synechocystis* sp. has also been reported [19]. The only slight decrease in anilofos concentration observed on day six in the control flask without *Synechocystis* sp. PUPCCC 64 cells (Fig. 5) indicated that anilofos is relatively stable and that its decline is mainly due to cyanobacterial uptake of anilofos and intracellular degradation.

### Optimal Conditions for Anilofos Removal

A number of factors such as pH, biomass and temperature have been shown to influence microbial degradation of chlorpyrifos [19], [67]–[69]. Thus, the effect of pH, biomass and temperature on anilofos removal by *Synechocystis* sp. strain PUPCCC 64 was studied. Maximum uptake of anilofos by PUPCCC 64 strain was observed at pH 8.0 (Table 2). Isolated and purified alkaline phosphatase from the cyanobacterium *Spirulina platensis* degraded chlorpyrifos at pH 10.0 [70]. *Anabaena inaequalis* degraded isoproturon at a fast rate at pH 5.5 [30]. Sorption of the herbicide paraquat by *Oscillatoria* sp. dominated cyanobacterial mat was not significant at pH 2.0–7.0 [71]. Thus different microorganisms have different pH optima for herbicides/pesticides degradation. Temperature is also reported to influence the rate of pesticide degradation [66], [67]. Thus, the effect of temperature on herbicide uptake by the test organism was studied. *Synechocystis* sp. strain PUPCCC 64 exhibited maximum uptake of herbicide at 30°C (Table 2). Our results are in agreements with the studies of Xu et al. [69] who reported maximum degradation of chlorpyrifos at 30°C by mixed cultures of bacterium *Serratia* sp. and fungus *Trichosporon*. To optimize biomass for maximum uptake of anilofos by the test organism, five biomass concentrations (25, 50, 100, 150, and 200 mg protein L^−1^) were selected (Table 2). *Synechocystis* sp. strain PUPCCC 64 exhibited increased anilofos uptake at a concentration of up to 100 mg L^−1^. Beyond this concentration anilofos uptake stagnated. This may be due to the fact that, at higher biomass loads, not enough herbicide molecules were available to saturate all the absorptive sites on the cyanobacterial cells. We earlier reported on the ability of *Synechocystis* sp. strain PUPCCC 64 to degrade chlorpyrifos at 100 mg protein L^−1^ biomass load [19]. With the current study we show for this same strain a similar capability for anilofos degradation. Such a display of dual tolerance against both a pesticide (chlorpyrifos) and an herbicide (anilofos) is at this time rather uncommon in cyanobacteria.

**Table 2:** Effect of pH, temperature, and biomass on anilofos uptake (mg L^−1^) by *Synechocystis* sp. strain PUPCCC 64.

pH				
5	6	7	8	9
2.5±0.01	3.3±0.02	3.7±0.02	4.2±0.03	3.2±0.01
**Temperature (°C)**				
20	25	30	35	40
2.5±0.01	2.9±0.01	3.8±0.02	3.4±0.01	1.8±0.01
**Biomass (mg L^−1^)**				
25	50	100	150	200
2.0±0.01	2.5±0.01	3.8^a^ ±0.03	3.7^a,b^ ±0.02	3.6^b^ ±0.01

Data in rows with same lowercase letter are not significantly different from each other at the 95% confidence level (p<0.05).

One parameter was changed at a time keeping other parameters (optimum value) constant.

Data are means of three observations ± SD.

Anilofos used during experiments: 10 mg L^−1.^

### Anilofos Mineralization and its Use as a Nutrient

**Figure 6:**
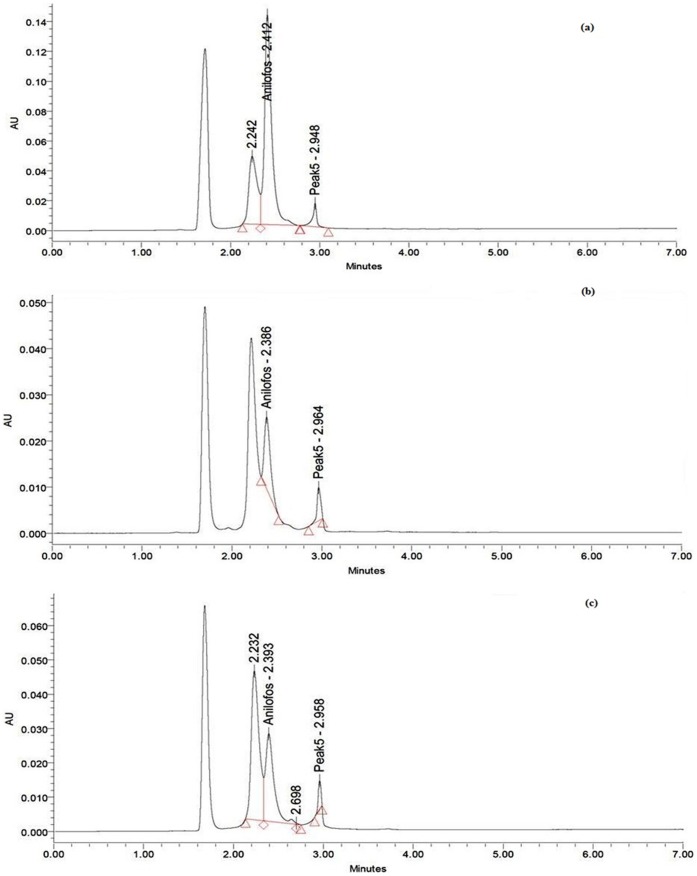
HPLC chromatogram showing the peaks of (a) standard anilofos, (b) cell extract and (c) supernatant. First large peak in the HPLC chromatogram represents solvent used to dissolve extracted anilofos.

**Figure 7:**
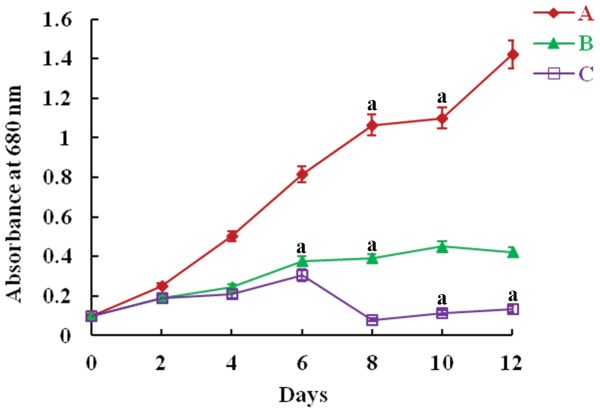
Growth of *Synechocystis* sp. strain PUPCCC 64 in basal medium (A) with 0.01 g K_2_HPO_4_ L^−1^, (B) with 2.5 mg anilofos L^−1^ without phosphate and (C) without phosphate. All presented data are the mean values of three independent experiments ± SD Data at different time intervals with same lower case letters are not significantly different from each other at the 95% confidence level (p<0.05).

The overall pattern of the HPLC chromatograms for biomass extract and supernatant were similar to pure anilofos used as standard (Fig. 6). No additional peaks were observed in HPLC chromatograms neither for biomass nor for supernatant (Fig. 6b and 6c). These results indicated that *Synechocystis* sp. strain PUPCCC 64 did not form any degradation products nor it released into the medium. Of the total 1000 µg anilofos (peak area 7.9×10^5^ AU (absorbance units), Fig. 6a) present in culture medium at the start of the experiment, only 69.7 (peak area 0.55×10^5^ AU, Fig. 3b) and 91.6 µg (peak area 0.73×10^5^ AU, Fig. 3c) herbicide was recovered from biomass and supernatant, respectively, after 4 days. From these results, it follows that nearly 839 µg anilofos seems to have been mineralized by the organism into CO_2_ and H_2_O. It is possible that *Synechocystis* sp. strain PUPCCC 64 uses the released phosphate component of anilofos as a nutrient. To test this hypothesis, the PUPCCC 64 strain was grown for 12 day in anilofos (2.5 mg L^−1^) phosphate deficient basal medium. Whereas strain PUPCCC 64 did not grow in phosphate deficient medium after day 4 it survived at a slow growth rate in presence of anilofos until day 12 (Fig. 7). This confirms that strain PUPCCC 64, in the absence of phosphate in the medium, used the phosphate component of anilofos as a nutrient. Various cyanobacteria and algae are able to degrade pesticides or herbicides and utilize the break-down products to sustain growth. Organophosphate pesticides malathion, monochrotophos and quinalphos metabolized by the cyanobacterium *Anabaena fertilissima* could be used as phosphorus source [72]. Phosphonate herbicide glyphosate was mineralized by cyanobacterium *Spirulina* spp. and used as phosphorus and nitrogen source [32]. A number of filamentous cyanobacteria and unicellular green algae (Chlorophyceae) were reported to degrade fenamifos pesticide into fenamifos sulfoxide as a primary product which was further transformed into fenamifos sulfoxide phenol [73]. And four cyanobacterial strains, *Anabaena* sp. PCC 7120, *Leptolyngbya boryana* PCC6306, *Microcystis aeruginosa* PCC7941 and *Nostoc punctiforme* PCC73102 were able to utilise the herbicide glyphosate as the sole phosphate source [31]. Limited data are available on the environmental fate of anilofos. Five reactions of anilofos degradation, namely hydrolytic cleavage of P-S-aryl linkage, N-dealkylation, O-demethylation, aryl hydroxylation and conjugation, were observed in the rat [74]. No reports indicating route(s) of anilofos degradation in microorganisms are available. Since *Synechocystis* sp. strain PUPCCC 64 grew in phosphate deficient anilofos supplemented medium, it is possible that this strain causes hydrolytic cleavage of P-S aryl linkage in anilofos releasing phosphate to be utilized as a nutrient. From the above discussion, it is clear that many environmental cyanobacteria and microalgae have developed a tolerance to otherwise toxic pesticides including herbicides and even are able to utilize the degradation products to sustain growth.

### Conclusions

We have demonstrated in this study that *Synechocystis* sp. strain PUPCCC 64 tolerated anilofos herbicide stress by increasing the level of antioxidant enzymes. Additionally, the strain PUPCCC 64 mineralized anilofos intracellularly and utilized released phosphate. This is the first report on anilofos degradation by a cyanobacterium. Since this strain has a potential to degrade both anilofos as well as chlorpyrifos, thus, it might be employed for bioremediation of these harmful chemicals from Indian paddy field ecosystems through biofertilizer programmes.
